# The multiple effects of fecal microbiota transplantation on diarrhea-predominant irritable bowel syndrome (IBS-D) patients with anxiety and depression behaviors

**DOI:** 10.1186/s12934-021-01720-1

**Published:** 2021-12-28

**Authors:** Hao Lin, Qingqing Guo, Zhiyong Wen, Songlin Tan, Jie Chen, Lijian Lin, Pengcheng Chen, Jianquan He, Jianbo Wen, Ye Chen

**Affiliations:** 1 Department of Gastroenterology, Fujian Provincial Hospital South Branch, No. 516, South, Jinrong Road, Cangshan District, Fuzhou, 350000 Fujian China; 2 Shengli Clinical Medical College, Fujian Medical University, No.134, East Street, Gulou District, Fuzhou, 350000 Fujian China; 3 Department of Gastroenterology, Integrative Microecology Center, Shenzhen Hospital, Southern Medical University, 1333 New Lake Road, Shenzhen, 518100 China; 4 Department of Intensive Medicine, The First Affiliated Hospital of Fujian Medical University, No.20, Chazhong Road, Taijiang District, Fuzhou, 350005 Fujian China; 5 Department of Gastroenterology, Affiliated Pingxiang Hospital, Southern Medical University, No. 8, Wugong Mountain Avenue, Development Zone, Pingxiang, 337055 Jiangxi China; 6 Department of Emergency, Fujian Provincial Hospital, Fujian Medical University, No.134, East Street,Gulou District, Fuzhou, 350000 Fujian China; 7 Department of Health Management, Fujian Provincial Hospital South Branch, No. 516, South Jinrong Road, Cangshan District, Fuzhou, 350000 Fujian China; 8 School of medicine, Xiamen University, Xiamen, China; 9 State Key Laboratory of Organ Failure Research, Guangdong Provincial Key Laboratory of Gastroenterology, Nanfang Hospital, Southern Medical University, Guangzhou, 510515 China

**Keywords:** Irritable bowel syndrome, Fecal microbiota transplantation, Anxiety and depression, Short-chain fatty acids, Metagenome

## Abstract

**Background:**

Anxiety and depression are complications in Irritable bowel syndrome (IBS) patients. In this study, we recruited 18 IBS patients with mild-modest anxiety and depression behaviors, and after the screening, we defined the FMT treatment group (n = 9) and the control group (n = 9). The IBS symptom severity scale (IBS-SSS), Hamilton Anxiety Rating Scale (HAM-A), Hamilton Depression Rating Scale (HAM-D), Irritable Bowel Syndrome Quality of Life (IBS-QOL) and Bristol stool scale (BSS) were evaluated one week before FMT (baseline), one-week-, one-month-, two-month-, and three-month-following FMT. Meanwhile, we determined the SCFAs in the patient’s feces and serum and continued the metagenomic analysis of the microorganisms in the patient’s feces.

**Results:**

The results showed that the patient’s anxiety and depression behavior gradually improved with FMT treatment. Moreover, the illness and quality of life had also been relieved significantly. The content of isovaleric acid and valeric acid was significantly reduced in the FMT group compared to the Col group. Metagenomic analysis showed that FMT treatment decreased the abundance of *Faecalibacterium, Eubacterium* and *Escherichia*. From KEGG functional analysis, we confirmed that the top five abundant pathways were “bacterial chemotaxis, “flagellar assembly”, “glycine, serine and threonine metabolism”, “apoptosis”, and “bacterial invasion of epithelial cells”.

**Conclusions:**

FMT treatment can effectively alleviate the anxiety and depression behaviors of IBS-D patients and reduce the IBS-SSS score, indicating that FMT can improve patients’ symptoms. The high throughput sequencing results show that *Bifidobacterium* and *Escherichia* play the most critical role in the formation and recovery of IBS-D patients. The GC/MS data indicated that faeces isovaleric acid and valeric acid might be more suitable as a metabolic indicator of IBS-D remission.

*Trial registration* ChiCTR, ChiCTR1900024924, Registered 3 August 2019, https://www.chictr.org.cn/showproj.aspx?proj=41676.

**Supplementary Information:**

The online version contains supplementary material available at 10.1186/s12934-021-01720-1.

## Background

In the past decades, the incidence and prevalence of Irritable bowel syndrome (IBS) have increased worldwide [1]. IBS results from a complex interplay between genetic, immunologic, microbial, and environmental factors, making the development of a simple and effective treatment a challenging task. According to the IBS patients’ predominant bowel habit, Rome IV categories were build-up for the classification of patients with IBS: diarrhea-predominant (IBS-D), constipation-predominant (IBS-C), mixed diarrhea/constipation (IBS-M), and unclassified (IBS-U). IBS-D may pose a greater diagnostic challenge than the other bowel habit types because patients with chronic or recurrent diarrhea need to consider celiac disease and inflammatory bowel disease [2]. With the deepening of research work on IBS, the progress of the brain-gut axis proved that gut microbes were closely related to emotions [3–5]. Anxiety/depression and IBS are highly prevalent and burdensome conditions, of which co-occurrence is estimated between 44 and 84% [6]. Liu et al. found that the fecal microbiota profiles in patients with depression were similar to that of IBS-D patients [7]. One hypothesis to explain its mechanism is that the mucosal immune barrier dysfunction induced by microbiota dysfunction leads to increased intestinal permeability, which may be an early step leading to the common pathogenesis of depression and IBS [8, 9].

Meanwhile, the primary metabolites of intestinal bacteria also play an essential role in the brain-gut axis. As we know, short-chain fatty acids (SCFAs), the main metabolites produced in the colon by bacterial fermentation of dietary fibers and resistant starch [10], are speculated to play a vital role in neuro-immunoendocrine regulation. Recent findings exhibited that SCFAs have important immunomodulatory functions. Substrate transporters like MCT1 and SMCT1 facilitate the absorption of SCFAs to promote cellular metabolism. Moreover, SCFAs may signal through cell surface G-protein coupled receptors (GPCRs), like GPR41, GPR43, and GPR109A, to activate signaling cascades that control immune functions [11]. Also, the SCFAs can affect cytokine production and migration, cytolytic activity, and epigenetic modulation [12].

As we know, the traditional method of treating IBS aims to reduce symptoms. However, there are many disadvantages and unclear effects in traditional treatment. The emergence of Fecal microbiota transplantation (FMT) treatment, focusing on the microbiome, becoming a fascinating area of research. Nowadays, the understanding of the effectiveness of FMT for IBS, especially the diarrhea-predominant type, is still in its infancy. Due to the differences in various experimental subjects, experimental conditions, and donor conditions, many medical experiments with conflicting results have been caused. Salhy et al. summarized why FMT treatment has different effects on improving the IBS symptoms in different queues, and they believe that FMT is an effective treatment for patients with IBS [13]. Nevertheless, this still needs more work to reveal its mechanism and effectiveness.

In this present study, we recruited IBS-D patients with mild-modest anxiety and depression behaviors, and after the screening, we defined the FMT treatment group (FMT) and the control group (Col). Follow-up was performed to determine the effectiveness of FMT and the improvement of patients’ emotion at one week before FMT, one-week-, one-month-, two-month-, and three-month-following FMT. Meanwhile, we determined the SCFAs in the patient’s feces and serum and continued the metagenomic analysis of the microorganisms in the patient’s feces. This research is expected to provide evidence for the effectiveness of FMT treatment and reveal the mechanism between FMT and emotions.

## Results

### Patient characteristics

The clinical characteristics of patients were shown in Table 1. The average ages of the Col group and T group were 50.44 and 44.33 (p > 0.05), respectively. At baseline, all patients have mild-modest anxiety and depression. Most of the Bristol stool forms of all patients were type 6 (n = 10, 5 in Col, 5 in T) or type 7 (n = 6, 4 in Col, 2 in T). The IBS-SSS ranged from 220 to 320 (avg 287.78), and the IBS-QOL ranged from 32 to 56 (avg 43.72) at baseline.

**Table 1:** Patient characteristics at baseline

Groups	Patients ID	Age	Gender	HAM-A	HAM-D	IBS-SSS	BSS	IBS-QOL
Col	1	69	Male	20.5	20	280	6	53
	2	59	Femal	20.5	21.5	220	6	40
	3	65	Male	17	20.5	320	6	40
	4	48	Male	14.5	20.5	320	7	42
	5	38	Male	23.5	22.5	300	6	56
	6	43	Femal	28.5	25.5	340	7	50
	7	50	Male	14.5	20.5	220	6	32
	8	42	Femal	18	25	260	7	48
	9	40	Femal	16.5	23.5	300	7	36
Avg.	–	50.44 ± 10.68	–	19.28 ± 4.30	22.17 ± 1.96	284.44 ± 40.86	–	44.11 ± 7.61
FMT	10	42	Femal	24.5	24	260	6	51
	11	35	Male	15	21.5	260	7	48
	12	39	Male	23	21.5	360	5	48
	13	63	Male	16.5	27.5	260	6	28
	14	47	Male	18.5	22	300	5	44
	15	39	Femal	17.5	22	320	6	40
	16	36	Femal	18	20.5	340	7	54
	17	58	Male	15.5	20	220	6	38
	18	40	Femal	16.5	22	300	6	39
Avg.	–	44.33 ± 9.31	–	18.33 ± 3.10	22.33 ± 2.11	291.11 ± 42.28	–	43.33 ± 7.53

## FMT treatment could relieve patient anxiety and depression

**Fig. 1:**
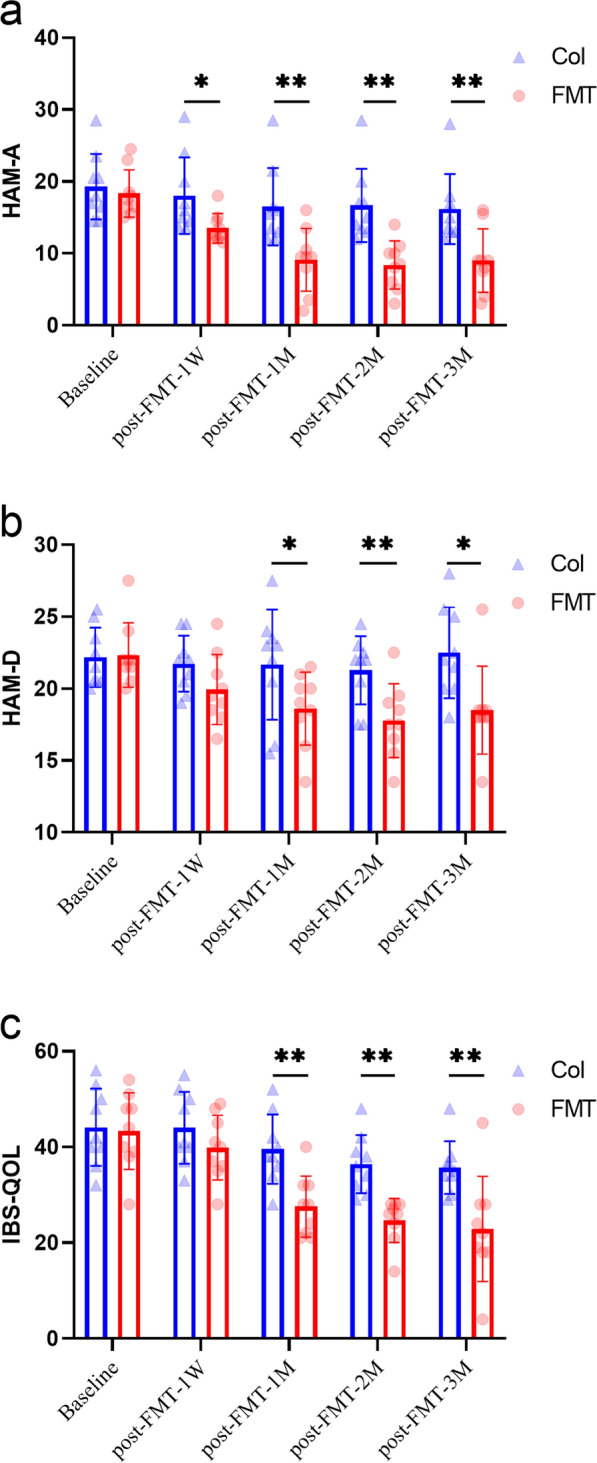
Effects of FMT on Hamilton Anxiety Rating Scale, Hamilton Depression Rating Scale and Irritable Bowel Syndrome Quality of Life in IBS-D patients with anxiety and depression. **a–c** shows the HAM-A, HAM-D and IBS-Qol indexes, respectively, of IBS-D patients in Col and FMT. The blue triangles represent the individuals of Col and the red dots represent the individuals of FMT. Bars indicate mean ± SEM. IBS-D, diarrhea-predominant irritable bowel syndrome; Col, Control group; FMT, FMT treatment group; HAM-A, Hamilton Anxiety Rating Scale; HAM-D, Hamilton Depression Rating Scale; IBS-QOL, Irritable Bowel Syndrome Quality of Life. The abscissa represents the sample collection time. All these evaluation indexes mentioned above were measured at one week prior to FMT (pre-FMT-1 W), one week following FMT (post-FMT-1 W), one month following FMT (post-FMT-1 M), two months following FMT (post-FMT-2 M), and three months following FMT (post-FMT-3 M). *, p < 0.05; **, p < 0.01

In this study, FMT treatment significantly alleviated patients’ anxiety and depression, which was mainly reflected in the continuous decrease of HAM-A and HAM-D with the increase of treatment time (Fig. 1a and b). The two groups showed significant differences in the first week after FMT treatment in HAM-A evaluation. When the FMT treatment time was prolonged to 1 month, the mean value difference between the two groups was remarkable in both HAM-A and HAM-D evaluation, which was maintained in the second and third months. In addition, in the follow-up process, we could observe the mood improvement of the FMT-treated patients. The evaluation of IBS-Qol showed that FMT could improve patients’ quality of life at the 1st, 2nd and 3rd month post-FMT-treatment (Fig. 1c). In summary, FMT treatment could relieve anxiety and depression after short-term treatment, and the longer the time, the better the effect. The improvement in the quality of life could also be observed after the FMT treatment.

### Remission of IBS symptoms by FMT

**Fig. 2:**
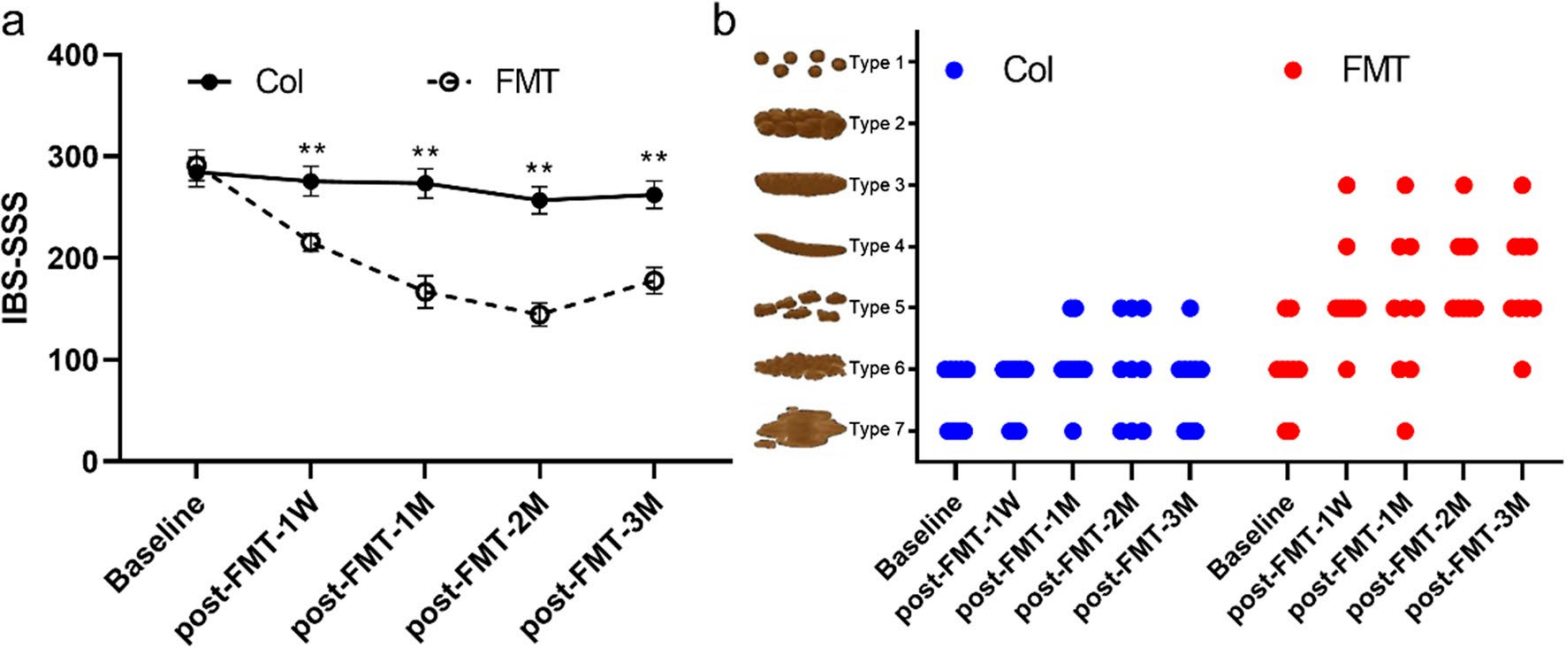
Effects of FMT on IBS symptom severity scale and Bristol stool scale in IBS-D patients with anxiety and depression. (**a**) The abscissa represents the sample collection time: one week prior to FMT (pre-FMT-1W), one week following FMT (post-FMT-1W), one month following FMT (post-FMT-1M), two months following FMT (post-FMT-2M), and three months following FMT (post-FMT-3M). Bars indicate mean +/− SEM. IBS-SSS, IBS symptom severity scale. (**b**) The Bristol stool scale of Col and FMT group. *, *p* < 0.05; **, *p* < 0.01

The IBS-SSS score of patients receiving fecal donor material decreased after FMT compared to baseline.The IBS-SSS score of patients in the FMT group was statistically lower than that of patients in the Col group at each time point after treatment. No significant difference in the IBS-SSS score was observed within the Col group between the different time points (Fig. 2a). In addition, FMT could also significantly improve fecal type (Fig. 2b). The stool types of most patients at baseline were between type 5 and type 7. However, after FMT treatment, a considerable number of patients had fecal type 3 or 4. In particular, there was no type 7 in the FMT group in the second and third months after completion of therapy.

### The FMT treatment caused the changes of SCFAs in feces and serum

**Fig. 3:**
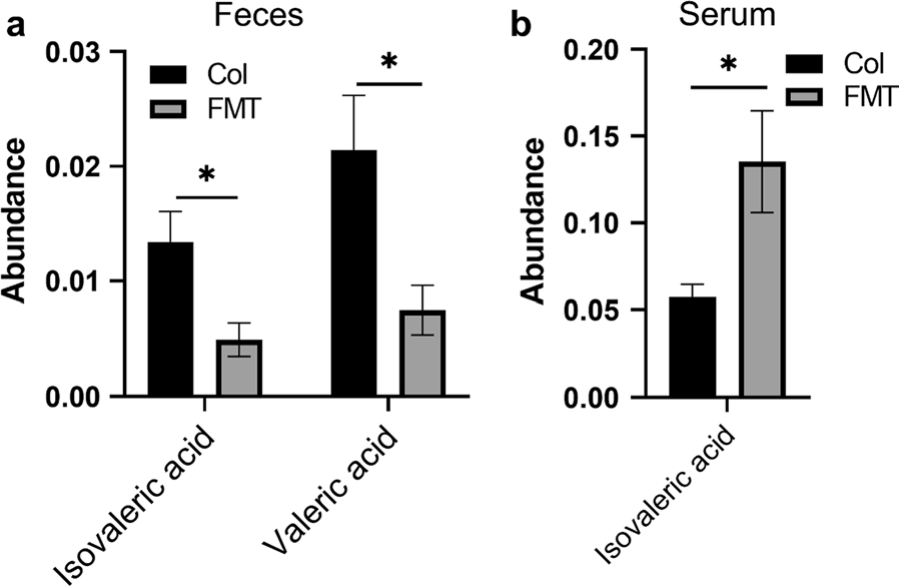
Effects of FMT on short-chain fatty acids (SCFAs) (**a**) and (**b**) shows the level of differential SCFAs in feces and serum, respectively. *, *p* < 0.05; **, *p* < 0.01

We collected the patients’ feces for SCFAs assay. There was no differential SCFAs found between the two groups at baseline. Nevertheless, as illustrated in Fig. 3a, the isovaleric acid and valeric acid levels were sharply reduced in the FMT group compared to the Col group at one-month post-FMT treatment. After KEGG enrichment analysis, we confirmed that isovaleric acid participates in the “Protein digestion and absorption” pathway (ko04974). Moreover, no significant difference in the concentration of SCFAs in serum was found between the two groups at baseline. However, the serum isovaleric acid level was significantly increased in the FMT group (Fig. 3b).

### The taxa of the fecal microbiota

**Fig. 4:**
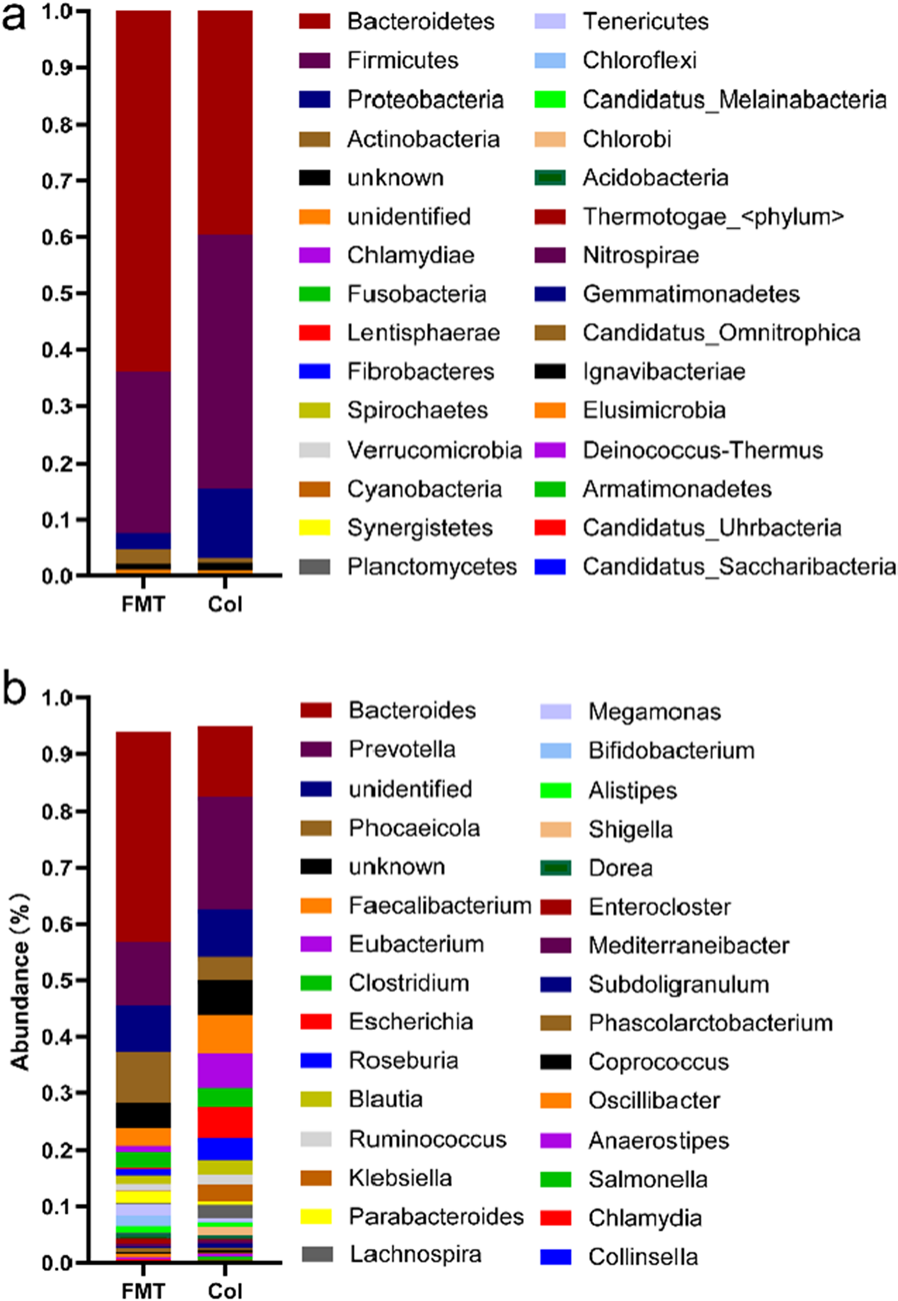
The taxa of the fecal bacteria. (**a**) and (**b**) indicate the bacteria taxa in Col and FMT group at the phylum and genus level, respectively. The stacking diagram shows the cumulative abundance of various bacteria

We found that Bacteroidetes and Firmicutes were the main phyla in both groups (Fig. 4a). At the genus level, the abundance of dominant genera, such as Bacteroides and Phocaeicola, were significantly increased by FMT treatment (Fig. 4b). Besides, the level of Bifidobacterium was also showed a significant increasing trend in the FMT group. Conversely, FMT treatment decreased the abundance of Faecalibacterium, Eubacterium and Escherichia (Fig. 4b).

### Top abundant pathways from KEGG functional analysis

**Fig. 5:**
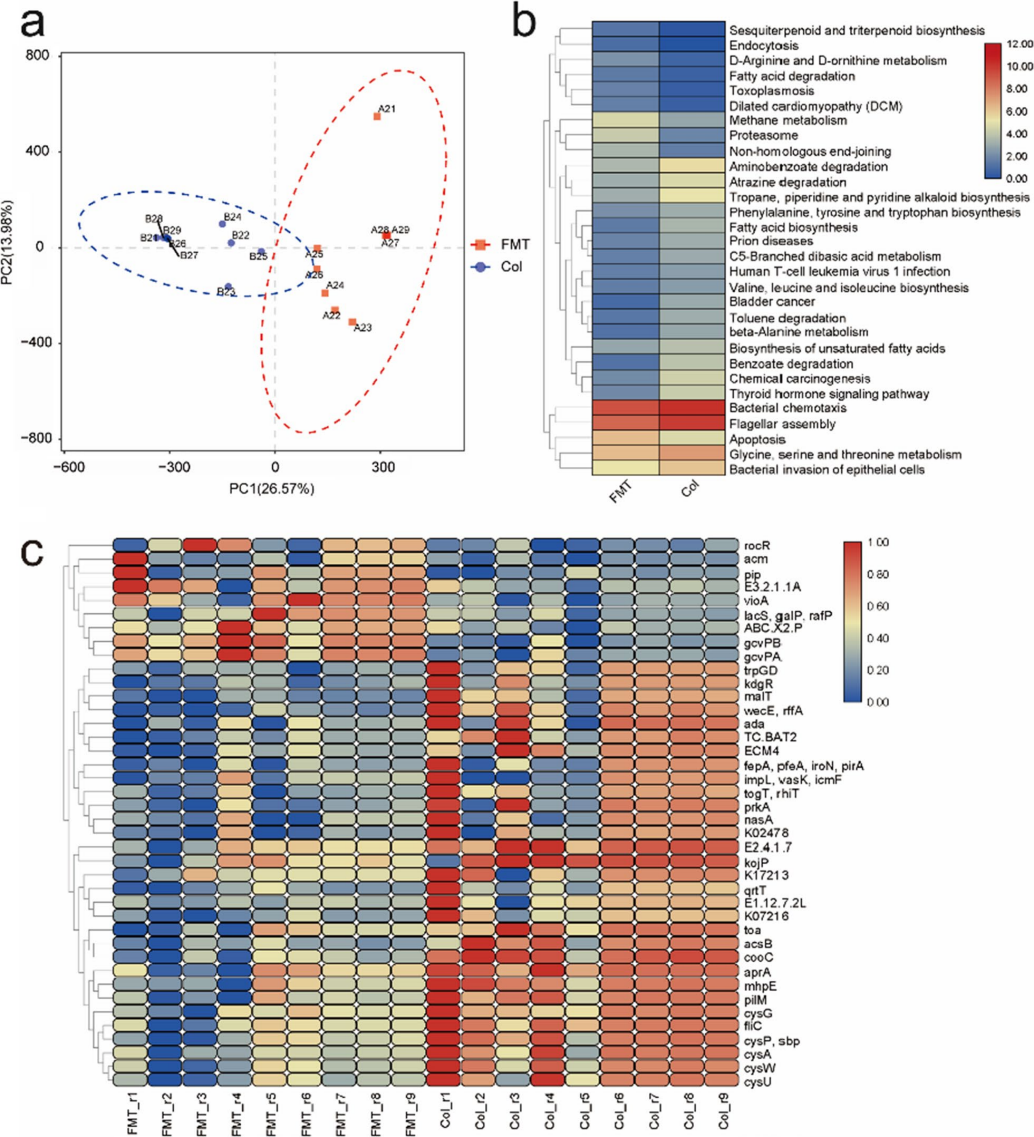
Results of KEGG functional analysis (**a**) PCA of the functional genes of the 18 IBS-D patients (PC1, principal component 1; PC2, principal component 2). Red boxes and blue dots represent samples in FMT and Col group, respectively. (**b**) The abscissa is the group name, the ordinate is the gene function, and the color depth represents the gene function abundance. The figure shows the top 30 KEGG pathways. (**c**) The heatmap shows the functional gene abundance of the top 40 genes. The color depth represents the gene abundance

We used the PCA to explore and visualize the difference between the Col and FMT groups. The PCA result showed that samples in both groups were evidently separated (Fig. 5a). Similarly, the apparent differential distributions of KEGG pathways between the two groups were observed (Fig. 5b). From KEGG functional analysis, we found that the top five abundant pathways were “bacterial chemotaxis, “flagellar assembly”, “glycine, serine and threonine metabolism”, “apoptosis”, and “bacterial invasion of epithelial cells”. We further screened out the top 40 most abundant genes, which were statistically different in the two groups (Fig. 5c), such as some *ABC transporter, malt, mhpE and acm*. In addition, we classified these genes according to the species and functions, and the result showed that most of these genes belonged to Escherichia coli. These genes mainly participate in the process of flagellin, collagen-binding protein, ferric enterobactin receptor and cellulose synthase (Additional file 1: Table S1).

## Resistance gene analysis based on the CARD database

**Fig. 6:**
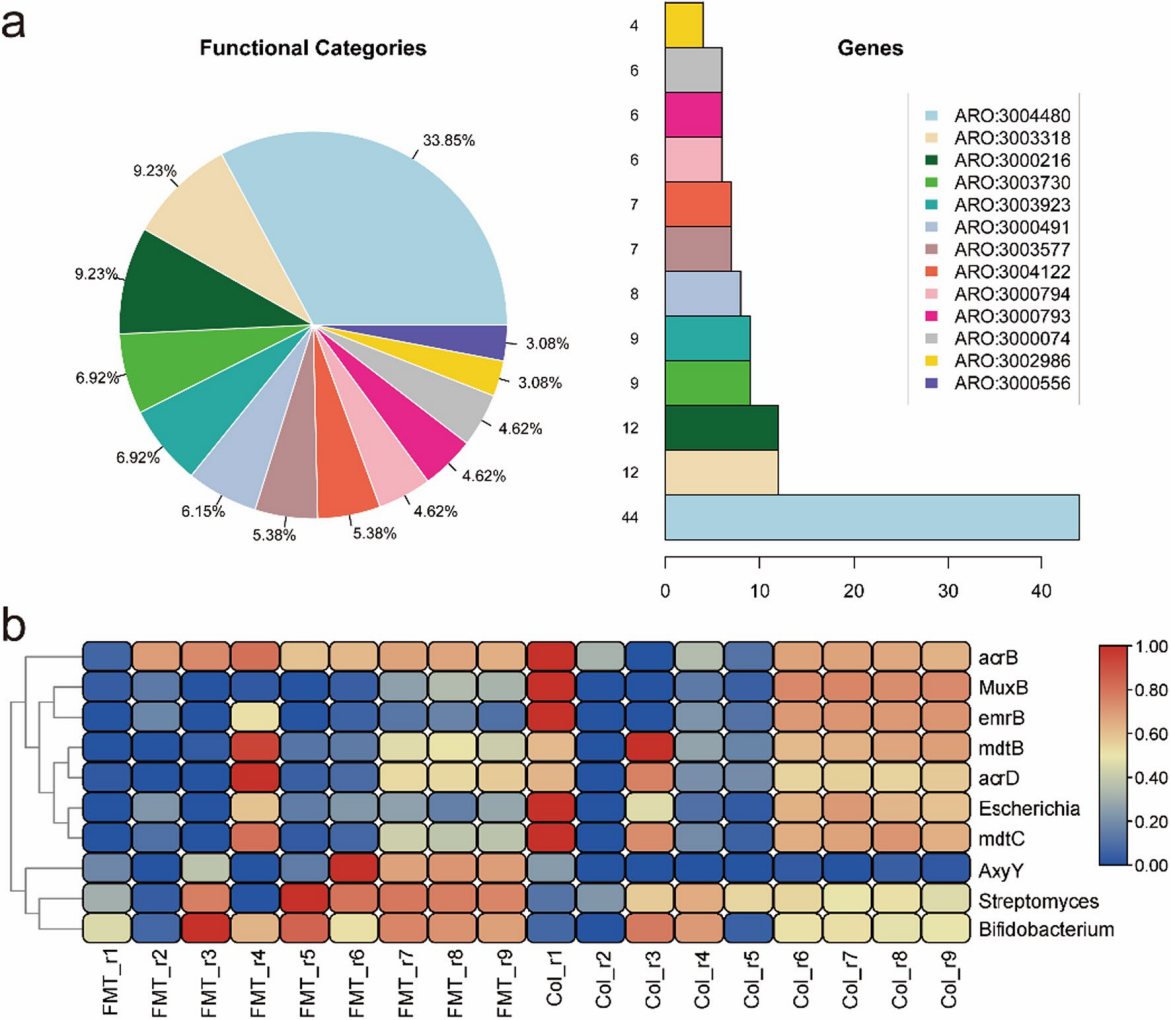
Resistance gene analysis base on the CARD database (**a**) The functional categories and genes enriched in CARD database. (**b**) The top ten genes (or bacteria) with significant differences between the two groups. The heatmap shows the functional gene abundance and the color depth represents the gene abundance

According to the result of resistance gene analysis, we classified all resistance genes. The result showed that the genes belonging to ARO:3,004,480 accounted for the largest proportion (Fig. 6a, Additional file 2: Table S2). The annotated taxonomy of ARO:3,004,480 was *Bifidobacterium adolescentis*. There were 12 genes classified to ARO:3,000,216 (*Escherichia coli*), ranking second in all classifications. The resistance gene analysis result identified the top ten genes (or bacteria) with significant differences between the two groups (Fig. 6b). In particular, the abundance of the feature gene of *Escherichia* was decreased significantly in the FMT group. However, the abundance of the feature gene of *Bifidobacterium* was significantly increased by FMT treatment.

## Discussion

Our results show that the FMT treatment could improve the IBS-D patients’ IBS-SSS scores and quality of life. Meanwhile, it could significantly reduce the scores of the HAM-A and HAM-D of those patients, which might maintain for some time. Studies on the brain-gut axis also showed that emotions are closely related to the intestinal flora and the environment of the intestine [14]. Intestinal microecological imbalance has been found to be closely related to IBS, inflammatory bowel disease, asthma, emotion (such as anxiety and depression) and other pathological conditions [15]. The results of this study show that FMT treatment could improve patients’ IBS-SSS scores and the quality of life. Meanwhile, FMT treatment greatly reduced the patient’s anxiety and depression behaviors after a short-term treatment and maintained a long time. There have been many precedents that have proved that FMT could improve the anxiety and depression-like behaviors of experimental subjects [16–19]. Another recent probiotic study that targeted patients with depression showed that subjective depression symptoms improved after probiotic administration and the serum high-sensitivity C-reactive proteins significantly decreased [20]. The benefits of the changes brought about by FMT to patients were obvious. Zhou et al. reported that transplantation of fecal microbiota, from normal mice to antibiotic-pretreated PD mice increased dopamine levels in the recipient PD mice, suggesting that gut microbiota contributed to the neuroprotection for PD [21]. There are evidences that dopamine plays an important role in anxiety and depression [22, 23]. Based on this, we speculate that FMT treatment could contributed to the neuroprotection and alleviate the anxiety- and depression-like behaviors of patients by regulating the dopamine levels. In addition, gut microbes affect the central nervous system by interacting with the vagus nerve [24]. As we know, the vagus nerve is composed of 80% afferent nerve fibers and 20% efferent nerve fibers. The afferent nerve fibers of the vagus nerve can be affected by the metabolites of the microbiota, and then bring these gut microbial signals back to the central nervous system [25]. As reported by Breit, the vagus nerve could be affected by long and short chain fatty acids both directly and indirectly, through cellular production of neurotransmitters [26]. Changes in enteric neuron activity perceived by the vagus nerve are essential for mediating satiety, stress, and mood [27, 28]. SCFAs, including acetic acid, propionic acid, butyric acid, isobutyric acid, valeric acid, and isovaleric acid, are end products from intestinal microbial fermentation. Previous studies reported that *Bifidobacterium* was positively correlated with isovaleric acid, and *Escherichia* was negatively correlated with isovaleric acid and valeric acid [29, 30]. In this present study, the fecal isovaleric acid and valeric acid were significantly reduced after FMT treatment (Fig. 3), which was correlated with the changes in intestinal flora abundance, such as the decline of *Escherichia* and the increase of *Bifidobacteria* (Fig. 4). As we know, valeric acid is commonly found in human feces, it is normal that it cannot be detected in the serum [31]. As the regulator, SCFAs might indirectly affect the anxiety and depression through sympathetic nerve. Also, the significant reduction of isovaleric acid and valeric acid in feces might be an obvious sign of the improvement of FMT treatment.

As we know, mucosa is one of the most important parts of the wall of intestine. The mucosa is composed of a single layer of epithelial cells and contains a large number of immune cells. The presence of the epithelial layer is essential for the normal function of the gastrointestinal tract. Meanwhile, the epithelium could effectively resist harmful macromolecules and microorganisms, which act as an important barrier to maintain intestinal health [32]. Impairment of the barrier function could increase permeability, which leads to the abnormal immune response to microorganisms, and luminal antigens had been proposed as an initiating factor in the pathogenesis of chronic human IBS [33, 34]. Previously research reported that one common feature of inflammation-associated microbiotas is increased levels of flagellin, which can occur owing to changes in species composition and/or microbial gene expression [35–39]. The link between elevated microbiota flagellin levels and intestinal inflammation is thought to involve flagellin’s ability to activate pro-inflammatory gene expression [40]. In the present study, many genes with significant abundance difference were enriched in “flagellar assembly” pathway, which might reflect enriched levels of motile bacteria that have high ability to penetrate the mucus layer that serves to protect the host against microbial onslaught [40]. Besides, we found that many genes with significant abundance difference were enriched in “bacterial chemotaxis, “glycine, serine and threonine metabolism”, “apoptosis” and “bacterial invasion of epithelial cells” pathways. These pathways were closely related to the interaction between epithelial cells and intestinal microorganisms, suggesting that FMT treatment had a positive effect on the reconstruction of intestinal mucosal barrier. Especially, compared with the Col group, FMT treatment significantly reduced the activity of “bacterial invasion of epithelial cells” pathway, indicating that FMT reduced the interaction between pathogenic bacteria (e.g., Shigella, Salmonella, Listeria) and epithelial cells, that is, it reduced the infection risk to a certain extent. According to the genes’ annotation, we found that genes related to carbohydrate metabolism (*malt*), catalytic activity (*mhpE*), amino acid utilization (*acm*) and transporters (ABC transporter) were the main functional genes affected by FMT treatment. In addition, most of these genes were found belong to *Escherichia coli*, which corresponded well with the results of bacterial taxa results. Based on this, we inferred that the changes in these genes are caused by FMT reducing the abundance of *Escherichia coli* and increasing *Bifidobacterium.* And the interaction between bacteria and intestinal epithelial cells might be the key to the pathogenesis of IBS-D.

## Conclusions

The FMT treatment could effectively reduce the IBS-SSS score of IBS-D patients and alleviate the anxiety and depression behaviors of these participants, indicating that the FMT therapy might improve the symptoms of IBS-D patients. The GC/MS data indicated that the levels of the isovaleric acid and valeric acid in feces might be suitable metabolic indicators of IBS-D remission. Additionally, the high throughput sequencing results showed that *Bifidobacterium* and *Escherichia* may play an important role in the formation and recovery of IBS-D patients. Genes like *ABC transporter, malt, mhpE, acm*, etc. played a key role in pathways like “bacterial chemotaxis, “flagellar assembly”, “glycine, serine and threonine metabolism”, “apoptosis” and “bacterial invasion of epithelial cells”. These pathways may be the main pathways for the interaction between intestinal bacteria and the host.

## Materials and methods

### Participants

To investigate the gut microbiota in IBS-D patients, we recruited a total of 463 patients with diarrhea-predominant IBS, who also had symptoms of anxiety and depression, from the Pingxiang People’s Hospital from Aug. 2019 to Mar. 2021. The enrollment criteria were as follows: ages 20 years or older; IBS-D assessed and diagnosed with the Rome III Diagnostic Criteria (Longstreth et al., 2006); Hamilton Anxiety Rating Scale (HAM-A) score ranges 14-24; Hamilton Depression Rating Scale (HAM-D) score ranges 20-34.

Exclusion criteria were as follows: had abdominal surgery; suffering from human immunodeficiency virus infection; suffering from kidney disease; abnormal liver function indexes; suffering from abnormal thyroid function; history of mental illness; suffering from active infection (excluding *Clostridium difficile* infection); pregnant woman; people who have taken probiotics, prebiotics, antibiotics orally in the past 2 weeks; cases who are participating in or participated in other clinical trials within 3 months.

Finally, 18 IBS-D patients with symptoms of anxiety and depression were included and randomly divided into two groups: the control (Col) and FMT treatment (FMT).

### Donor

The donor used in this study was screened according to the guidelines for FMT donors in 2019 [41]. He was a healthy 36-year-old male, no smoking and drinking, not taking any medication regularly, no class A or class B infectious diseases or infection history and had a normal body mass index. He did not take antibiotics within three months, and had no history of gastrointestinal diseases and mental illness.

Preparation of intestinal bacteria: donor feces were collected and diluted with 500 ml of 0.9% normal saline. Then, the intestinal bacteria were extracted automatically by an intestinal bacteria extractor (Guangzhou chengge Biotechnology Co., Ltd.). The extracted bacterial solution was then centrifuged at 4000R/min for 10 min, and the supernatant was discarded to retain the sediment. After the precipitate is fully stirred, it is divided into capsules in equal quantities, and stored in the refrigerator at -80 ℃.

### Experimental design and sample collection

All the subjects were randomly divided into two groups, 9 in each group. For group FMT, all the nine included patients received FMT treatment (oral administration) from May 2019 to December 2019. The patients took the intestinal flora capsules 3 times in total, once every other day, 30 capsules each time. For group Col, the patients took the same amount of blank capsules. At the same time, patients’ psychiatric symptoms were evaluated by Hamilton Anxiety Rating Scale (HAM-A, https://dcf.psychiatry.ufl.edu/files/2011/05/HAMILTON-ANXIETY.pdf) and Hamilton Depression Rating Scale (HAM-D, https://dcf.psychiatry.ufl.edu/files/2011/05/HAMILTON-DEPRESSION.pdf) method [42] during the FMT process to analyze the impact of FMT on psychiatric symptoms, and to seek the relationship between microbiota composition and psychiatric symptoms. During the same period, statistics and analysis of Bristol stool scale (BSS) [43, 44], IBS symptom severity scale (IBS-SSS), and Irritable Bowel Syndrome Quality of Life (IBS-QOL) [45, 46] were performed to evaluate the effectiveness of FMT treatment. Briefly, the questionnaire of IBS-SSS is meant to register complaint levels related to gastrointestinal symptoms in the form of four questions: (a) Do you suffer from abdominal pain? b) Do you currently suffer from abdominal distention? (c) How satisfied are you with your bowel habits? (d) Please indicate on the line (visual analog scale) below how much your IBS is affecting or interfering with your life in general? A total of 500 points can be reached. A score of up to 75 points is considered as control, 75–175 points as mild IBS, 175–300 as moderate IBS, and more than 300 as severe IBS. For IBS-QOL, the score ranged from 0 to 100 points and a higher score indicated a better QOL. The score contained 34 questions from 8 aspects: dysphoria, interference with activity, body image, health worry, food avoidance, social reaction, sexual concern and relationship. Stool consistency was assessed by the 7-point Bristol stool scale, a higher score indicating a softer stool. Stool frequency was defined as stools per day. All the evaluation indexes mentioned above were measured at one week prior to FMT (pre-FMT-1 W), one week following FMT (post-FMT-1 W), one month following FMT (post-FMT-1 M), two months following FMT (post-FMT-2 M), and three months following FMT (post-FMT-3 M).

Stool and serum samples were collected one week prior to FMT (pre-FMT-1 W) and one month following FMT (post-FMT-1 M). Serum samples were used to detect Short Chain Fatty Acids (SCFAs) through GC/MS technology. The stool sample was divided into two parts, one was used to detect SCFAs, and the other part was used for metagenomic analysis.

### Sample preparation and GC/MS analysis

Stool and serum samples were pretreated according to Zhang report [47]. Briefly, adding 10 mM sodium bicarbonate solution to 1 g samples; then, adding external standard mixture, tert-butyl methyl ether and HCl solution to the supernatant after centrifugal extraction; finally, centrifuge again to retain the supernatant for GC/MS analysis. Each sample were prepared and processed three times.

The SCFAs in the samples were analyzed as free acid form using a Supelco Nukol column 30 m length, 0.25 mm internal diameter and 0.25 μm of film thickness with the temperatures program as follows: Initial temperature of 40 °C was held for 1 min, then it was increased to 150 °C at 30 °C/min, finally grow up to 220 °C at 20 °C/min. A 1 µL aliquot of extracted sample was injected in splitless mode (splitless time 1 min) at 250 °C, while the transfer line temperature was 280 °C. The used carrier gas was helium and its flow rate maintained at 1 mL/min for whole run time. The MS acquisition was carried out in single ion monitoring by apply a proper dwell time (20 ms for each ion monitored) to guarantee a detection frequency of 4 cycle/s. The quantitative determination of SCFAs in each sample was carried out by the ratio between the area abundance of the analytes with the area abundance of respective labeled internal standard (isotopic dilution method). The value of this ratio was named peak area ratio (PAR) and it was used as abundance of each analyte in the quantitative evaluation. The detailed steps were listed in Additional file 3: Table S3.

### Metagenomics analysis

The fecal DNA was extracted using the QIAamp Fast DNA stool minikit (Qiagen, Hilden, Germany) according to the manufacturer’s instructions. The DNA was first fragmented (using covaris M220). Then, the NEBNext Ultra DNA library prep kit was used to construct the sequencing libraries. The prepared libraries were sequenced by an illumina HiSeq4000 platform with 2 × 150 paired-end configuration.

The metagenome data analysis pipeline was shown in Additional file 4: Figure S1. Briefly, the sequence data was firstly evaluated by Trimmomatic software, and the sequencing adapters and sequences with low quality scores were trimmed. High quality short reads were then assembled by the MEGAHIT software (v1.0.6) with default parameters [48]. For functional annotation, MetaGeneMarK was employed to predict the ORFs of the assembled contigs [49]. The predicted ORFs were grouped using CD-HIT with the coverage over 90% and minimum identity 95%, and finally the non-redundant gene set was obtained [50].

We used BLAST to search for the protein sequences (translated from ORFs) of the predicted Genes in KEGG databases (http://www.genome.jp/kegg/), with *e*-value < 1e^−5^. The genes with KEGG annotation were assigned into KEGG pathways. For taxonomic assignment we used the Diamond software [51]. Then, based on gene abundance, PCA, PCOA, and PLS-DA analysis were performed by our internal scripts. The difference between different groups were calculated by Kruskal Wallis test. For all analysis details, please refer to the report by Song et al. [52].

### Statistical analysis

Statistical analysis was performed using the SPSS 22 statistical software. Means and standard error of means for the different treatments were calculated and differences between means were determined by t-test on HAM-A, HAM-D, IBS-QOL and IBS-SSS scores. In all cases the level of statistical significance was P < 0.05.

## Supplementary Information

**Additional file 1: Table S1.** Gene annotation results.

**Additional file 2: Table S2.** Genes annotation based on the CARD database.

**Additional file 3: Table S3.** GC/MS detailed experimental steps.

**Additional file 1: Figure S1.** Metagenome analysis pipeline.

## Data Availability

We have uploaded the data to the SRA and Metabolights. The data deposited in the SRA and Metabolights is publicly available.

## Abbreviations

HAM-A: Hamilton Anxiety Rating Scale
HAM-D: Hamilton Depression Rating Scale
IBS-QOL: Irritable Bowel Syndrome Quality of Life
IBS: Irritable bowel syndrome
IBS-D: Diarrhea-predominant
IBS-C: Constipation-predominant
IBS-M: Mixed diarrhea/constipation
IBS-U: Unclassified
SCFAs: Short-chain fatty acids
GPCRs: G-protein coupled receptors
FMT: Fecal microbiota transplantation
Col: Conventional treatment group
ChiCTR: Chinese Clinical Trial Registry
BSS: Bristol stool scale;
IBS-SSS: IBS symptom severity scale
IBS-QOL: Irritable Bowel Syndrome Quality of Life
SCFAs: Short Chain Fatty Acids
PAR: Peak area ratio
