# Supplementary material for: The multiple effects of fecal microbiota transplantation on diarrhea-predominant irritable bowel syndrome (IBS-D) patients with anxiety and depression behaviors
Source: Microb Cell Fact. 2021 Dec 28;20:233. doi: 10.1186/s12934-021-01720-1 (PMC8715582; doi:10.1186/s12934-021-01720-1)
Supplement: Supplementary file 3 — Additional file 3: Table S3. GC/MS detailed experimental steps. [file 12934_2021_1720_MOESM3_ESM.docx]

Table S3: GC/MS detailed experimental steps

1. Chemicals and reagents

Methyl tert-butyl ether (MTBE) were purchased from CNW (CNW Technologies, Germany). MilliQ water (Millipore, Bradford, USA) was used in all experiments. All of the standards were purchased from CNW (Beijing) or aladdin(Shanghai). The stock solutions of standards were prepared at the concentration of 1 mg/mL in MTBE. All stock solutions were stored at -20 °C. The stock solutions were diluted with MTBE to working solutions before analysis.

2. Sample preparation and extraction

20 mg of fecal samples were accurately weighed and placed in a 2 ml EP tube. 1 mL of phosphoric acid (0.5% v/v) solution and a small steel ballwere added to the EP tube. The mixture wasgrinded for 10s,three times, then vortexed for 10 minutes and ultrasonicated for 5 minutes. 0.1 mL of supernatant was added to 1.5 mL centrifugal tube after the mixture was centrifuged at 12000 rpm for 10 minutes at the temperature of 4°C. 0.5 mL MTBE (containing internal standard) solution was added to the centrifugal tube. The mixture was vortexed for 3 minutes and ultrasonicated for 5 minutes. After that, the mixture was centrifugedat 12000 rpm for 10 minutes at the temperature of 4°C. The supernatant was collected and used for GC-MS/MS analysis.

3. GC/MS analysis

Agilent 7890B gas chromatograph coupled to a 7000D mass spectrometer with a DB-FFAP column (30 m length × 0.25 mm i.d. × 0.25 μm film thickness, J&W Scientific, USA) was employed for GC-MS/MS analysis of SCFAs. Helium was used as carrier gas, at a flow rate of 1.2 mL/min. Injection was madein the split mode and the injection volume was 2 μL. The oven temperature was held at 90°Cfor 1 min, raised to 100°C at a rate of 25°C/min, raised to 150°C at a rate of 20°C/min,hold on 0.6 min, raised to 200°C at a rate of 25°C/min, hold on 0.5min, after running for 3min. All samples were analyzed in multiple reaction monitoring mode. The injector inlet and transfer line temperature were 200 °C and 230 °C, respectively.
