# Supplementary figures and images for: The multiple effects of fecal microbiota transplantation on diarrhea-predominant irritable bowel syndrome (IBS-D) patients with anxiety and depression behaviors
Source: Microb Cell Fact. 2021 Dec 28;20:233. doi: 10.1186/s12934-021-01720-1 (PMC8715582; doi:10.1186/s12934-021-01720-1)

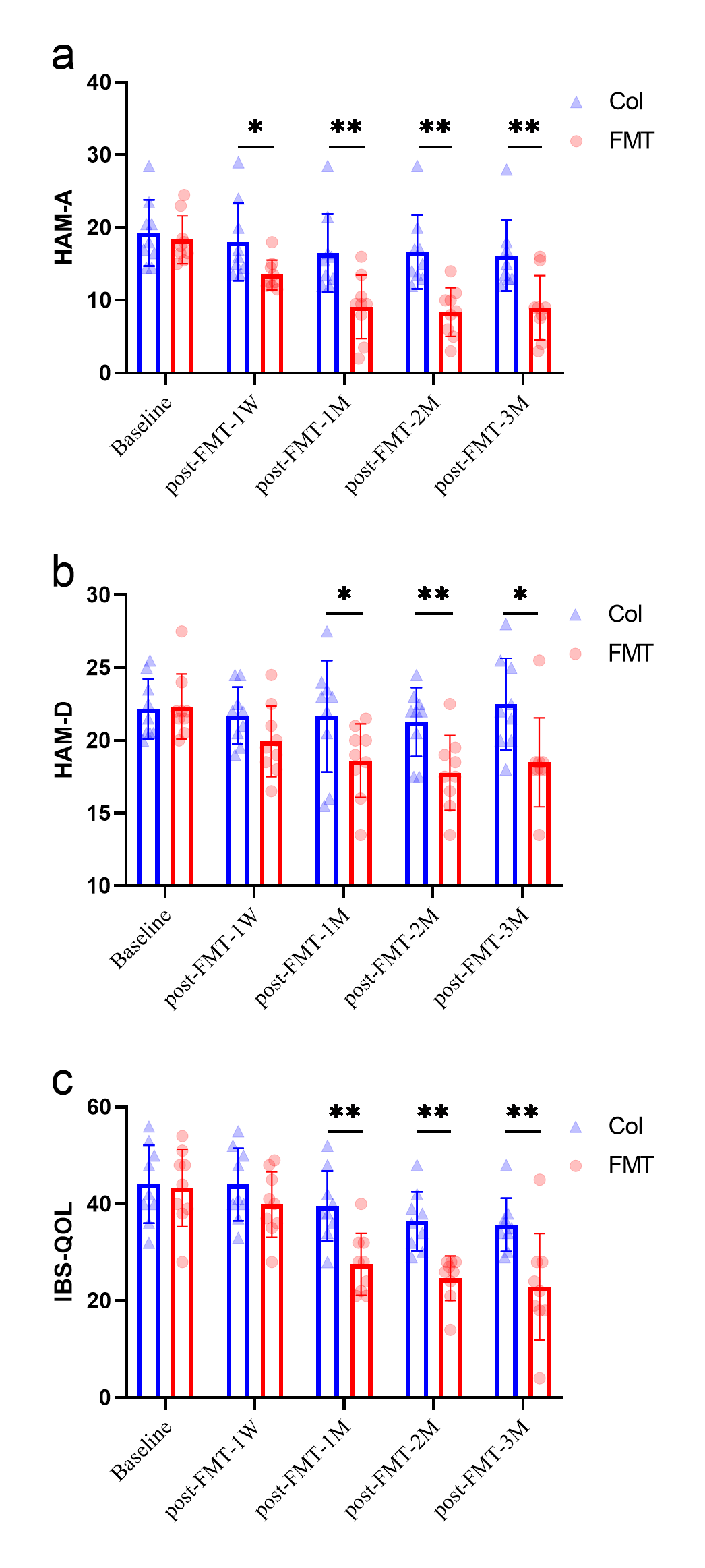

Supplement: Supplementary file 4 — Additional file 1: Figure S1. Metagenome analysis pipeline. [file 12934_2021_1720_MOESM4_ESM.tif]
